# Targeted and Sequential Cryoneurolysis Improves Gait After Botulinum-Toxin Unresponsiveness in Post-Stroke Spasticity: A Laboratory-Verified Case

**DOI:** 10.3390/neurolint18010013

**Published:** 2026-01-07

**Authors:** Frédéric Chantraine, José Alexandre Pereira, Céline Schreiber, Tanja Classen, Gilles Areno, Frédéric Dierick

**Affiliations:** RehaLAB, Centre National de Rééducation Fonctionnelle et de Réadaptation—Rehazenter, 1, Rue André Vésale, 2674 Luxembourg, Luxembourg; frederic.chantraine@rehazenter.lu (F.C.); jose.pereira@rehazenter.lu (J.A.P.); celine.schreiber@rehazenter.lu (C.S.); tanja.classen@rehazenter.lu (T.C.); gilles.areno@rehazenter.lu (G.A.)

**Keywords:** hemiplegia, spastic muscles, cryosurgery, rehabilitation, walking

## Abstract

**Background:** Chronic post-stroke spasticity often limits gait despite best-practice botulinum-toxin intramuscular injections (BTIs), whose benefit is constrained by short duration, dose ceilings, and tachyphylaxis. Cryoneurolysis (CNL) induces a reversible axonotmesis with preserved endoneurium, potentially providing longer tone reduction with fewer adverse effects, but its impact on whole-gait quality and its compatibility with implanted functional electrical stimulation (FES) remain poorly documented. **Case presentation:** A 43-year-old man, 12 years after right middle cerebral artery stroke, walked independently with an implanted common peroneal FES system but complained of effortful gait with left-knee “locking” and drop foot without FES. Multiple BTI series to triceps surae and quadriceps yielded only transient benefit. Two ultrasound-guided CNL sessions targeted tibial (soleus, medial gastrocnemius) and femoral (rectus femoris, vastus intermedius) motor branches. Quantitative gait analysis and fine-wire electromyography (EMG) were performed at baseline, 6 weeks after each CNL, and at 6 months, with and without FES. CNL produced immediate and sustained reductions in triceps surae and quadriceps overactivity, resolution of genu recurvatum, normalization of stiff-knee gait, improved ankle dorsiflexion, and increased swing phase knee flexion (>50°). Gait Deviation Index rose from 69 to 80 and Gillette Gait Index decreased by more than 50%, with preserved strength and without adverse events. **Conclusions:** Targeted, sequential CNL of tibial and femoral motor branches can safely deliver durable, clinically meaningful gait improvements when BTI has reached its ceiling and can act synergistically with implanted FES. Quantitative gait analysis and EMG sharpen clinical decision-making in spasticity management.

## 1. Introduction

Restoring community ambulation remains a central yet elusive goal of chronic stroke rehabilitation. For most survivors, residual dorsal flexor weakness and plantar flexor hypertonia provoke equinus and quadriceps overactivity limits knee flexion and ultimately increases the risk of falls. Current best-practice guidance assigns botulinum-toxin intramuscular injection (BTI) first-line status but also concedes three hard constraints: (i) a median efficacy window of only 12–16 weeks, (ii) a regulatory ceiling of 400 U Onabotulinum-A per 12 weeks, and (iii) a progressive tachyphylaxis attributed to the development of neutralizing antibodies [1,2,3]. Chemical neurolysis with phenol or alcohol can extend tone reduction, yet carries a risk of dysaesthesia and myofibrosis [4]. Given these constraints, residual spasticity often persists despite care, and patients accept sub-optimal gait.

Cryoneurolysis (CNL) offers a mechanistically distinct alternative. A −35 °C Joule–Thomson effect produces a Sunderland grade II lesion (axonotmesis) with intact endoneurium, permitting orderly axonal regrowth (about 1 mm day^−1^) and effectively eliminating neuroma risk [5]. Small observational series now place CNL among one of the few evidence-based options beyond BTI [4], yet its impact on whole-gait quality has never been quantified, and its compatibility with implantable foot-drop stimulators is unknown.

We present, to our knowledge, one of the first cases in which stepwise, sequential ultrasound-guided CNL of tibial (first) and femoral (second) motor branches was followed by six-month gait improvement corroborated by quantitative gait analysis, without compromising the added value of an implanted common peroneal functional electrical stimulator. The report adheres to the CARE guidelines [6].

## 2. Case Report

**Figure 1:**
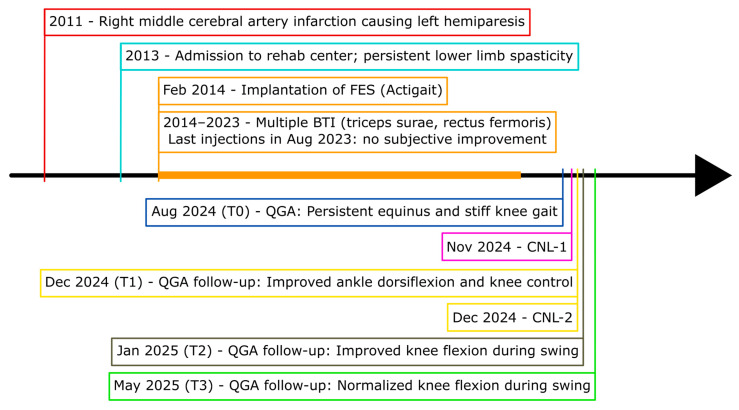
Long-term clinical timeline and procedural milestones. Chronological overview from index stroke (age 31) to the 6-month post-CNL-1 assessment. BTI = botulinum-toxin injection (five gastrocnemii/soleus (100 U) and five rectus femoris (200 U) series, 2014–2023); FES = implantable common peroneal functional electrical stimulation system (ActiGait, implanted in 2014); CNL-1 = tibial-motor-branch CNL (Week 0); CNL-2 = femoral-motor-branch CNL (Week 6).

A 43-year-old male, 12 years post-right middle cerebral artery infarction, walked independently with an ActiGait (Ottobock, Duderstadt, Germany) functional electrical stimulation (FES) system but reported slow, effortful gait and left-knee “locking”. Multiple triceps surae and quadriceps BTI series gave only transient relief. Without FES, he showed drop foot, stiff-knee gait, and recurvatum, matching pattern IIb of the chronic stroke typology [7]. Given the unsatisfactory BTI response, we proposed precision CNL targeting selected motor branches. Two sessions were conducted (Figure 1): the first addressed the soleus and medial gastrocnemius branches of the tibial nerve (CNL-1), and the second targeted the rectus femoris and vastus intermedius branches of the femoral nerve (CNL-2).

Throughout his follow-up [pre-intervention (T0), six weeks after each CNL session (T1, T2), and six months after first CNL intervention (T3)], the patient underwent quantitative gait analysis (QGA), with and without FES, during 10 m straight-line walking at self-selected speed. When FES was applied, stimulation parameters (pulse width, frequency, amplitude, and trigger timing) were standardized at T0 and kept identical across all subsequent acquisitions (T1–T3). This standardization was intended to isolate CNL-related changes from potential variability induced by stimulation settings.

Passive ankle dorsiflexion range of motion (ROM) was assessed in supine position (with knee flexed/extended) with a goniometer, while muscle strength was evaluated with the Medical Research Council (MRC) scale [8]. Spasticity of the quadriceps was rated using the Modified Ashworth Scale (MAS) and triceps surae using the ankle Tardieu Scale (TS) at fast speed (V3) [9]. The clinical evaluation was carried out by the same evaluator (FC).

QGA included kinematics and kinetics acquired via a 10-camera optoelectronic motion capture system (Oqus4, Qualisys, Göteborg, Sweden, 200 Hz) and two force plates (OR6-5, AMTI, Watertown, MA, United States of America (USA), 2000 Hz). Five gait cycles per condition were recorded and averaged. Dynamic muscle activity was measured (TeleMyo Desktop DTS, Noraxon, Scottsdale, AZ, USA, 2000 Hz) using ultrasound-guided intramuscular fine-wire electromyography (EMG) targeting triceps surae (soleus, gastrocnemius medialis and lateralis) and quadriceps (rectus femoris, vastus intermedius and medialis). EMG recordings were band-pass filtered (60–500 Hz) and full-wave rectified. EMG interpretation focused on visually, within-muscle changes detected in burst timing and relative amplitude across T0 and T2. In T0, two baseline knee patterns (recurvatum or flexion), likely reflecting fluctuations in hemiparetic motor control and compensatory strategies, were observed. Time was normalized to the gait cycle with toe-off alignment.

**Figure 2:**
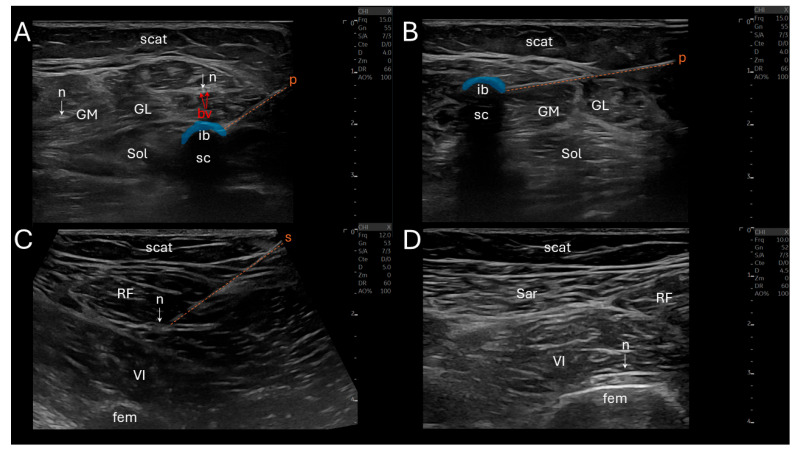
Ultrasound-guided CNL of tibial and femoral motor branches. (**A**) Transverse view showing the cryoprobe (p) (orange dotted line, freezing phase of one of the soleus (Sol) motor branch with the hypoechoic ice ball (ib) in blue and a posterior shadow cone (sc) at the probe tip, and adjacent gastrocnemii medialis (GM) and lateralis (GL) respective nerves (n, white arrows); (**B**) view of the freezing phase of one of the GM motor branch with ib (in blue) and sc; (**C**) view of the stimulation needle (s and orange dotted line) on one of the rectus femoris (RF) motor branch (n, white arrow); (**D**) view of one of the vastus intermedius (VI) motor branch (n, white arrow), and adjacent sartorius (Sar) and RF. bv (red arrows) = blood vessels, fem = femur, scat = subcutaneous adipose tissue.

CNL was performed using a N_2_O-based system (Metrum Cryoflex, Warsaw, Poland) delivering cryogenic temperatures through a 14G percutaneous cryoprobe (Figure 2). The target motor nerve was cooled to approximately −30 °C to −40 °C to induce a controlled, reversible axonotmesis. All CNL procedures were conducted under sterile conditions, without sedation or general anesthesia, and under real-time ultrasound guidance using a linear 10–15 MHz probe (Logiq E10, General Electric, Boston, MA, USA).

The seven procedural steps of CNL (total duration ≈ 1 h) were the following: (i) a sonographic landmarking in a transverse view, (ii) the introduction of an insulated stimulation needle (22G, 10 cm) via an in-plane technique, directing it towards the nerve trajectory, while avoiding vascular structures, (iii) a nerve stimulation initiated at 2 mA, gradually reduced to 0.5 mA to ensure close proximity to the motor fascicles (muscle contraction remains visible), (iv) needle-to-cryoprobe exchange, (v) a reconfirmation of neurostimulation twitch via the cryoprobe prior to freezing, (vi) the CNL phase (first cycle: 2 min continuous freezing, thawing: 45 s, second cycle: another 2 min freezing) creating an ellipsoidal anechoic ice ball and its posterior acoustic shadow cone at the tip of the cryoprobe, and (vii) a post-lesion stimulation was performed to confirm temporary denervation (absence of evoked muscle contraction at 1 mA). If the nerve still responded to this stimulation, a supplemental freezing of 2 min was applied.

Stimulation of the soleus was performed proximally, 2–3 cm distal to the popliteal fossa, in two points, medially and laterally close to the tibial nerve. Stimulation of the gastrocnemius medialis was performed slightly more proximally, intramuscularly. The rectus femoris and vastus intermedius stimulations were also performed proximally, distal to the inguinal ligament, laterally close to the femoral nerve, and the stimulation of the vastus intermedius required a deeper insertion.

**Figure 3:**
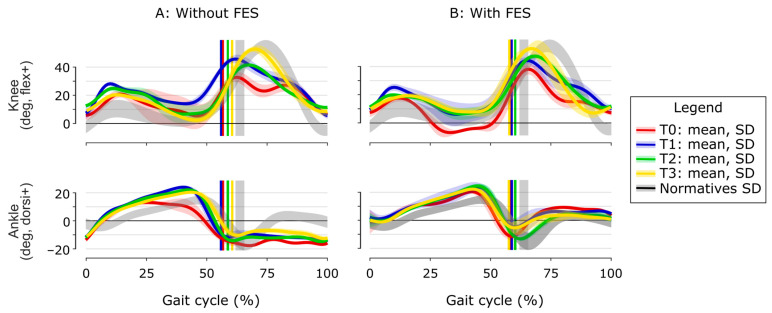
Sagittal plane kinematics for knee and ankle joints without (A) and with (B) FES at baseline (T0) to follow-up (T3). Mean ± SD ankle (**bottom**) and knee (**top**) joint angles; the gray band (one SD around mean) is the laboratory adult normative data (n = 40). Vertical bars mark toe-off. (**A**) Without FES: At baseline (T0, red) findings confirmed: ankle plantar flexion during swing (dorsiflexion −12.9°) and during stance (no heel strike and lack of first rocker), high variability of the knee behavior during stance (recurvatum or flexion) explained by a lack of sufficient knee control and a stiff-knee gait pattern with knee flexion of 32.8° during swing, corresponding to moderate severity (braked-knee type) [12]. (**B**) With FES: At T0, dorsiflexion improved during swing (9.3°), but knee hyperextension (genu recurvatum) worsened (−6.8°) during stance. CNL-1 (T1, blue) increased peak stance phase dorsiflexion by +11° and eliminated mid-stance recurvatum; CNL-2 (T2, green) added +13° of swing phase knee flexion, normalized (more than 40° [12]) the abnormal “braking” of the knee at around 60% of gait cycle. Six months after CNL (T3, yellow), knee kinematics have improved further toward normative values (swing phase knee flexion 53.5°) while ankle has remained stable.

Baseline clinical examination (T0, Table 1) revealed normal passive ankle dorsiflexion ROM and preserved sensory function. MRC indicated normal knee extensors’ strength and reduced ankle plantar flexors’ strength. Spasticity was observed for quadriceps and triceps surae. TS revealed dynamic ankle clonus triggered at −30°. Without FES, kinematics during swing (Figure 3, red) shows ankle plantar flexion, and a stiff-knee gait pattern. The knee kinematics showed marked stance phase variability (important standard deviation), with a knee sometimes flexed, sometimes extended, varying from one walking cycle to another. GGI and GDI scores confirmed a global pathological gait. FES improved dorsiflexion during swing (9.3°), but increased knee hyperextension (genu recurvatum) (−6.8°). Gait velocity was 0.92 ± 0.05 m/s without FES and 0.97 ± 0.04 with FES.

After the CNL-1 (T1), without FES (Figure 3A, blue), ankle dorsiflexion improved during stance phase: peak dorsiflexion increased from 13.1 ± 1.9° to 24.0 ± 0.8° in the second rocker, with better knee control resulting in less variability and decreased knee hyperextension. During swing, peak knee flexion improved from 32.8° to 45.7°, despite persistent ankle plantar flexion. With FES (Figure 3B, blue), and during swing, ankle dorsiflexion improved and knee flexion remained above 40°, and during stance, genu recurvatum disappeared. The patient reported improved walking comfort and confidence, particularly during transitional movements and turning. GGI and GDI scores (Table 1) mirrored the kinematic gains: GGI dropped in the no-FES and FES conditions, while GDI rose in both test conditions. Spasticity of the triceps surae was reduced, with dynamic ankle clonus triggered at −5°. Gait velocity was stable, 0.88 ± 0.07 m/s without FES and 1.00 ± 0.03 with FES.

After the CNL-2 (T2) of the femoral motor branches (Figure 3A, green), ankle kinematics remained stable, with preserved improvements from CNL-1 (blue) with and without FES. Knee kinematics showed an increase in peak knee flexion during swing from 44.3° without FES to 47.4° with FES (Figure 3B, green). Therefore, the stiff-knee gait pattern was, by definition [12], completely corrected in the two conditions, since the peak knee flexion in swing was over 40°. GGI remained clearly below baseline and GDI stabilized (Table 1), indicating that the overall gait pattern, although slightly regressed without FES, was still markedly closer to normal in both test conditions (with and without FES) compared to T1. No spasticity was observed for quadriceps. Gait velocity was stable, at 0.92 ± 0.06 m/s without FES and 1.03 ± 0.03 with FES.

At follow-up (T3, Figure 3, yellow), ankle kinematics remained stable, while knee kinematics improved further and were very close to the normative values, with a knee flexion in swing of 53.5°. The GGI and GDI improvements were consolidated (Table 1).

**Figure 4:**
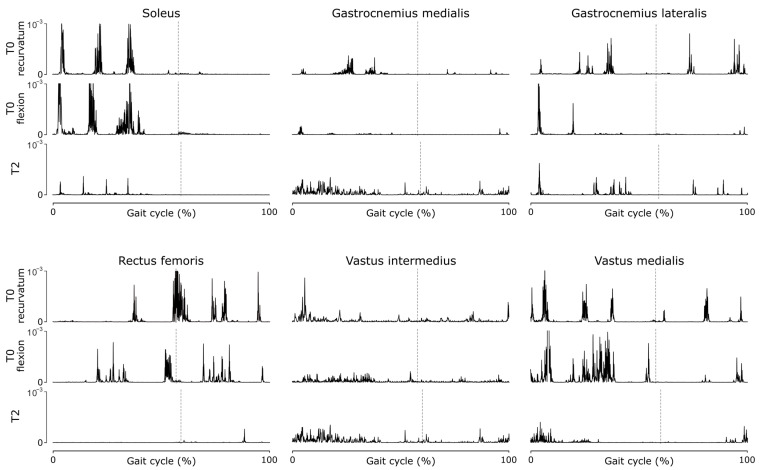
Rectified electromyography patterns at baseline (T0) for two knee kinematic strategies (recurvatum, flexion) and after CNL-2 (T2). Patterns are shown for left triceps surae (soleus, gastrocnemius medialis, and lateralis), and left quadriceps (rectus femoris, vastus intermedius, and medialis) during level walking at self-selected speed. Rows per muscle from top to bottom: T0—recurvatum, T0—flexion, T2. Time is normalized as % gait cycle. Vertical dashed lines mark toe-off. Patterns are identically scaled within each muscle.

Fine-wire EMG at T0 (Figure 4) showed two types of behaviors related to the two different knee kinematic strategies (recurvatum or flexion) in stance phase, with greater quadriceps activity when the knee is flexed. The triceps muscles exhibit hyperactivity during stance phase, contributing to the recurvatum, and the quadriceps muscles, especially the RF head, exhibit hyperactivity in the pre-swing and early swing, causing stiff knee. After the two stages of CNL (T2), fine-wire EMG revealed a substantial decrease in soleus and rectus femoris activity throughout the gait cycle, and no change in gastrocnemii and vastus intermedius activities.

The patient tolerated both CNL interventions well. There were no reported adverse events or unexpected reactions, no signs of neuropathic pain, dysesthesia, or muscle weakness, and no functional decline or compensatory gait deviations. The patient completed all follow-up assessments and remained adherent to gait training with FES and routine rehabilitation. The patient’s perspective was the following: *“After the last injections of botulinum toxin, I felt like nothing was improving anymore. My leg remained stiff and heavy, especially around my ankle and my knee. I started to lose confidence in walking, even with FES. When the CNL was proposed, I didn’t really know what to expect, but the procedure was quick, and I felt no pain at all after the technique. A few minutes later, I could really feel the difference. Walking became easier, and the knee didn’t lock as much. I feel more stable, especially when turning or walking longer distances. For the first time in a long time, I’m walking without constantly thinking about how to avoid falling.”*

## 3. Discussion

When compared with BTI, a practical benefit of CNL is its longer therapeutic horizon. Across mixed etiologies, clinical series summarized in a recent review report tone reduction that persists during 6–17 months in 60–70% of treated nerves, with only rare sensory sequelae [4]. By contrast, pooled randomized control trial data place the median functional benefit of BTI at 12–16 weeks despite repeat dosing [1,2,3], while chemical neurolysis (alcohol and phenol) offers variable 3–6-month relief at the cost of neuritis and myonecrosis [4]. The immediate onset due to the conduction block and longer therapeutic duration of CNL compared to BTI Wallerian degeneration abolishes pathological firing, yet the preserved endoneurial scaffolding requires several months for axonal regeneration and myelin remodeling [5]. In contrast to BTI, which reduces muscle overactivity primarily via neuromuscular junction blockade, motor-branch CNL predominantly interrupts efferent motor conduction (a reversible axonotmesis). Any concomitant afferent modulation is theoretically possible in mixed fascicular structures but was not assessed here and should be interpreted cautiously. Together, these mechanisms yield four clinical advantages: (i) fewer treatment visits are necessary; (ii) no cumulative-dose ceiling or antibody risk exists; (iii) a stepwise therapeutic approach is feasible; and (iv) enhanced synergy with adjunct technologies is possible: the multi-month antagonist quieting seen here, expanded the effectiveness window of the implanted foot-drop stimulator—something that a short-acting BTI could probably not achieve.

The present case extends the emerging CNL literature in four ways. First, it is consistent with the reversible axonotmesis model established histologically [5]. The large fall in EMG power, the parallel drop in MAS scores, and the preservation of voluntary strength jointly indicate that CNL silenced the targeted motor branches but did not definitively denervate it. This distinguishes cold-induced block from phenol neurolysis, whose unpredictable myotoxicity can blur the boundary between paresis and tone reduction [4]. Second, the kinematic and composite gait data demonstrate that neural silencing, translated into functionally relevant, whole-gait change. At six months, the GDI had climbed from 69 to 80, placing the patient within two SD of normative adult gait; concurrently, the GGI fell by more than 55%. Both shifts exceed the adult minimal detectable differences reported by [10,11]. A GDI of 80 also lies at the upper boundary of the “independent community ambulator” corridor identified by [2], linking laboratory gain to real-life walking ability. Third, the study is the first to show that CNL and an implanted ankle/foot dorsiflexors stimulator can act synergistically. Activating the FES device reduced GGI by a further 23–40%, implying that debulking antagonist tone enlarges the biomechanical “window” in which a neuro-prosthesis can operate. This interaction is clinically relevant, as many chronic stroke survivors who reach the therapeutic ceiling of BTI subsequently rely on orthotic or neuroprosthetic aid. Fourth, the pairing of CNL with full-wave rectified EMG patterns and global gait indices offers a reproducible evaluation framework. Unlike isolated spasticity scores, the combined approach bridges changes in motor-unit firing, joint kinematics, and integrated gait quality, aligning with current calls for precision and multi-scale outcome measurement in post-stroke rehabilitation [1,13,14]. As expected after tibial-branch CNL, plantar flexor strength transiently decreased (MRC 3/5 to 2/5) before returning to baseline at 6 months; importantly, this did not translate into functional deterioration and likely contributed to improved tibial forward-progression (2nd rocker) and reduced recurvatum.

In this single-case report, EMG was included as mechanistic corroboration rather than a primary quantitative endpoint. Accordingly, we pre-specified a qualitative, within-subject comparison focused on clinically interpretable features (phase-dependent timing referenced to gait events and changes in burst amplitude) between baseline and the post-intervention state. This approach was complemented by convergent evidence from clinical measures, sagittal kinematics, and global gait indices, strengthening causal attribution without requiring amplitude normalization.

Several practical considerations may help clinicians define candidacy, anticipate limitations, and optimize the implementation of motor-branch CNL in post-stroke spasticity. Procedural costs vary across health systems and formal cost-effectiveness analyses are not yet available. The technique requires specific training in ultrasound-guided peripheral nerve identification and motor-branch stimulation mapping and should be embedded in a structured post-procedure physiotherapy program to consolidate newly available motor patterns. CNL targets dynamic, neurally mediated overactivity; therefore, fixed contractures (e.g., structural equinus or fixed knee deformity) may limit the achievable biomechanical change. Because only motor branches were targeted, no sensory disturbance occurred in this patient; nonetheless, pre-existing peripheral neuropathy (e.g., diabetic neuropathy) may reduce predictability and is a relative contraindication. Very high spasticity scores combined with restricted passive ROM may indicate fixed stiffness and reduce responsiveness. There is no strict age cut-off; candidacy is guided by goals, comorbidity burden, and safe participation. Cognitive status does not alter the physiological response, but the ability to engage with rehabilitation and safety instructions influences functional gains.

The report is not without limitations: single-case design, unblinded assessors, and six-month follow-up constrain external validity. Nevertheless, the effect sizes surpass accepted minimal detectable changes for GDI (around 5 points) [11]. Future controlled series should benchmark CNL against repeat BTI cycles, include dynamometry to track re-innervation, and incorporate patient-centered metrics such as the Modified Gait Efficacy Scale [15]. Moreover, a 6-month time point coincides with the early phase in which axonal regeneration may begin after a Sunderland grade II lesion; longer follow-up is needed to characterize durability and potential waning of effect.

## 4. Conclusions

This case provides mechanistic, biomechanical, and functional evidence suggesting that image-guided CNL is a viable, device-compatible option when the “BTI ceiling” has been reached, and it illustrates how QGA, including sagittal kinematics, rectified EMG, and holistic gait metrics, can sharpen clinical decision-making in spasticity care.

## Figures and Tables

**Table 1:** Clinical examinations and quantitative gait metrics across all study visits (T0 to T3). T0: baseline; T1: 6 weeks after CNL-1; T2: 6 weeks after CNL-2; T3: 6-month follow-up after CNL-1 (and 18 weeks after CNL-2). ROM: passive joint range of motion, MRC: Medical Research Council strength grade [8], MAS: Modified Ashworth Scale, TS: Tardieu Scale [9], GGI: Gillette Gait Index [10], GDI: Gait Deviation Index [11]. Values are mean ± SD of five gait cycles (gait metrics) or single measurements (clinical variables). Boldface highlights changes exceeding published minimal detectable differences (≥5 GDI points, ≥30% GGI) compared to T0.

			T0	T1	T2	T3
Clinical Variables						
ROM (°)	Ankle (dorsiflexion)	Knee extended	10	15	10	15
		Knee flexed	15	15	20	15
MRC	Knee	Extensors	5	4	4	4
	Ankle	Plantar flexors	3	2	2	3
Spasticity	Knee (MAS)	Quadriceps (Ely test)	1+	1	0	0
	Ankle (TS)	Triceps surae	4(−30°)	1(−5°)	1(−5°)	2(−5°)
Gait metrics without FES						
	Ankle (°)	ROM 1st rocker	0.0 ± 0.0	0.0 ± 0.0	0.0 ± 0.0	0.0 ± 0.0
		Max in 2nd rocker	13.1 ± 1.9	24.0 ± 0.8	22.1 ± 0.7	20.5 ± 0.5
		Max dorsiflexion in swing	−12.9 ± 2.5	−9.1 ± 1.7	−11.5 ± 1.6	−7.0 ± 2.4
	Knee (°)	Extension in stance	5.2 ± 8.8	5.6 ± 2.3	6.6 ± 6.3	2.3 ± 2.0
		Flexion in swing	32.8 ± 3.4	45.7 ± 2.9	41.5 ± 1.2	52.8 ± 2.2
		ROM	34.8 ± 7.6	41.7 ± 4.7	36.4 ± 5.0	51.3 ± 1.8
	Velocity (m/s)		0.92 ± 0.05	0.88 ± 0.07	0.92 ± 0.06	0.98 ± 0.04
	Index	GGI	361.3 ± 25.8	**211.8 ± 57.1**	308.5 ± 62.3	**161.8 ± 24.9**
		GDI	69.8 ± 2.1	**76.1 ± 1.4**	74.4 ± 1.9	**80.1 ± 1.4**
Gait metrics with FES						
	Ankle (°)	ROM 1st rocker	0.0 ± 0.0	2.6 ± 2.0	2.3 ± 2.0	1.4 ± 1.5
		Max in 2nd rocker	20.4 ± 1.3	23.8 ± 3.4	24.5 ± 1.6	22.3 ± 1.0
		Max dorsiflexion in swing	9.3 ± 2.7	6.7 ± 4.5	4.4 ± 1.2	3.8 ± 3.0
	Knee (°)	Extension in stance	−6.8 ± 2.5	6.2 ± 8.3	6.4 ± 4.2	7.1 ± 3.5
		Flexion in swing	38.2 ± 3.7	44.3 ± 2.3	47.4 ± 1.7	53.5 ± 3.5
		ROM	47.0 ± 3.1	42.6 ± 8.7	41.7 ± 3.4	51.7 ± 3.0
	Velocity (m/s)		0.97 ± 0.04	1.00 ± 0.03	1.03 ± 0.03	0.94 ± 0.06
	Index	GGI	328.8 ± 52.0	**187.9 ± 25.2**	**185.2 ± 22.9**	**121.5 ± 30.8**
		GDI	69.7 ± 0.5	**77.4 ± 3.0**	74.6 ± 3.2	**80.2 ± 7.5**

## Data Availability

The original contributions presented in this study are included in the article. Further inquiries can be directed to the corresponding authors.
